# Static and Dynamic Activity Detection with Ambient Sensors in Smart Spaces

**DOI:** 10.3390/s19040804

**Published:** 2019-02-16

**Authors:** Sagar Shelke, Baris Aksanli

**Affiliations:** Electrical and Computer Engineering, San Diego State University, San Diego, CA 92182, USA; sshelke@sdsu.edu

**Keywords:** smart space, human activity detection, non-intrusive, ambient, big data analysis, machine learning

## Abstract

Convergence of Machine Learning, Internet of Things, and computationally powerful single-board computers has boosted research and implementation of smart spaces. Smart spaces make predictions based on historical data to enhance user experience. In this paper, we present a low-cost, low-energy smart space implementation to detect static and dynamic human activities that require simple motions. We use low-resolution (4 × 16) and non-intrusive thermal sensors to collect data. We train six machine learning algorithms, namely logistic regression, naive Bayes, support vector machine, decision tree, random forest and artificial neural network (vanilla feed-forward) on the dataset collected in our lab. Our experiments reveal a very high static activity detection rate with all algorithms, where the feed-forward neural network method gives the best accuracy of 99.96%. We also show how data collection methods and sensor placement plays an important role in the resulting accuracy of different machine learning algorithms. To detect dynamic activities in real time, we use cross-correlation and connected components of thermal images. Our smart space implementation, with its real-time properties, can be used in various domains and applications, such as conference room automation, elderly health-care, etc.

## 1. Introduction

Smart spaces have gained significant attention over the last several years due to advancements in the sensor technology, decreasing cost of hardware and ease of deployment. These spaces combine small and efficient hardware with data management mechanisms to provide solutions in various domains including health-care, wellness, education, etc. Smart spaces differ from traditional environments because of constant interactions between the users and sensing elements. One important research topic within smart spaces is human activity detection, due to its applications in robotics and human computer interaction (HCI). Detecting human activities accurately and in real time is a challenging problem for several reasons [1]. There are several methods to perform activity detection: using multimedia-sources (such as audio/video), wearable devices (smart watches, wristbands, etc.), and ambient sensing. Every method has its advantage and disadvantage based on its use cases. For example, despite having high accuracy, multimedia-based solutions can lead to privacy issues, i.e., users might not be willing to be identified in a video or audio recording for activity detection. Similarly, wearable device-based methods can provide customized solutions but also might lead to discomfort issues, negatively affecting the feasibility of the study. Ambient sensing, on the other hand, relies on sensors that provide only ambient information about the environment. It does not have any privacy or discomfort issues, but requires careful thinking in terms of sensor placement and data analytics. Ambient sensing to detect human activities uses one or more sensors of same/different modality. Each sensor has sensing noise and needs to be handled before output is fed to a decision-making system such as machine learning models, or an actuation unit.

Smart spaces are usually shared by multiple people. These shared spaces commonly suffer from a lack of reliable data metrics for an effective resource allocation and the ability to predict future changes in resource needs. The ability to recognize a person’s behavior and activities in real time plays a crucial role in providing useful data about the environment and the changes to be observed in the environment. Facilities management can use passive data gathering (no human intervention) to create more informed decisions about where to effectively allocate staff, what portions of their facilities are underutilized, addressing security concerns, and when and where to allocate expensive resources such as power dedicated to HVAC (heating, ventilation, and air conditioning) systems [2,3], or making educated guesses about the near-future availability of different rooms. Furthermore, real-time human activity detection and prediction can be used to effectively allocate resources to increase user comfort in the commercial settings such as conference rooms in offices [4], study rooms as well as in households [5]. Success of personal-assistive automation units can also be greatly improved by the ability of the automation unit (such as a robot) to understand the user’s activities and serve them accordingly [6]. The rapid aging of the population problem around the world revolves around these issues and has attracted ambient assisted living (AAL) as a potential solution [7], where human activity detection is an integral part.

In this work, we focus on detecting human activities that involve simple movements, such as sitting, standing, moving left/right, etc. We divide these simple human activities into static activities and dynamic activities. Static activities are those activities where the person is steady with respect to sensor setup in the environment. Standing still, sitting on the chair, sitting on the ground, and laying on the ground are the static activities considered in our environment. Dynamic activities are those activities where the person is moving continuously with respect to sensor setup in the environment. Moving to the left, moving to the right, moving towards the sensor, and moving away from the sensor are the dynamic activities modeled in our environment.

We model and implement a novel scheme for real-time and non-intrusive human activity detection. Use of low resolution (4 × 16) thermal sensors for data collection reduces computational complexity and removes any privacy concern (i.e., the users in the environment cannot be identified). We show that the feed-forward neural network algorithm results in the best average accuracy of 99.96% for static activity detection. We use the method of connected component labeling to detect forward–backward dynamic activities with 85.79% accuracy. Cross correlation of each frame with a center frame detects left–right dynamic movement with 100% accuracy. We also analyze the time overhead of our method, and observe that we can identify a method with high accuracy and low model train and test overhead (e.g., in our work, the random forest algorithm provides the best trade-off). This way, our implementation effectively recognizes human activities in real-time without compromising user identities or comfort.

## 2. Related Work

The human activity detection problem is solved in different ways in the literature. Activity detection has usually been performed using audio/video surveillance because the multimedia data provide very accurate representations of human activities [8]. Another method to obtain activity detection is using wearable devices [9]. This method has gained a lot of attention due to wearable devices becoming widely-available and popular among people. In this section, we are going to review the existing human activity detection methods in different categories, and then discuss how our method and setup is different than the previous studies.

### 2.1. Multimedia-Based Activity Detection

Multimedia-based human activity detection generally focuses on using images or videos. These methods have been popular because the data provide significant information about the human activity, increasing the activity detection accuracy. The video-based detection is very common as continuous activity demonstration is available in video data [10]. It is also possible to detect the activities from streaming real-time videos [11]. Authors of [12,13] used a static camera to capture and classify user activities, such as walking, standing, and sitting, in real time. In another work, Zhao et al. [14] used human shapes from video frames, and Wang et al. [15] used R-transform to detect static and dynamic human activities. Other efforts include activity detection using RGB-D (an RGB-D image is a a combination of a red-green-blue image and its corresponding depth image) images/videos [8], reducing the data size and making the identification of the user difficult.

Camera based activity detection methods are susceptible to variation in light intensity and variation in the background. Moreover, there is a trade-off between sensing efficiency (increases with camera resolution) and computational overhead for real-time applications. Vaizman et al. [16] analyzed data from accelerometer, magnetometer, gyroscope, audio and location sensors, collected using smartphones and smartwatches. Models trained on their dataset could recognize 15 human activities with an average F-1 score of 60% and balanced accuracy of 87%. Use of data from camera and audio increases classification accuracy but poses a question on user privacy. In contrast, identifying a user in a low-resolution thermal sensor is impossible, which maintains user privacy in smart spaces. In addition, static placement of these sensors within the environment remove user dependency. Basu et al. [17] used a thermal sensor to detect left–right and up-down human movement. Two more very relevant studies, by Shah et al. [18] and Akula et al. [19], have proposed activity detection using infrared (IR)-based video snippets, where each snippet is 0.5 s long, and images. These studies apply a convolutional neural network on IR data. Although the studies provide useful insight and are similar to ours, there are some basic differences: (1) the reported accuracy is around 85% (whereas, we can obtain up to 99% accuracy), (2) the study does not provide computational overhead analysis (e.g., train/test execution time analysis), and (3) the study applies only a neural network-based method (whereas we provide results with a range of other methods, enabling comparison among these methods in terms of accuracy and computational overhead).

For both image and video-based detection, the raw data is usually pre-processed to extract features, using some transformation algorithms (e.g., discrete Fourier transform, scale invariant feature transform, etc.). Then, the processed data is fed into some classification algorithms to label the activities using hidden Markov models, support vector machines, neural networks, etc. [20]. To aid the work in this domain, researchers have released large-scale databases that include video-based activity benchmarks [21]. The advantage of image/video-based activity detection is the potential high accuracy. In addition, since the activities can be observed in images/videos, it is easier to obtain the ground truth before constructing a model. In contrast, there are several disadvantages, including (1) the size of data: image/video data may require significant storage, (2) computation overhead: image/video data need pre-processing, making it difficult to obtain real-time results, and (3) privacy issues: the user is completely or partially exposed in image/video data.

### 2.2. Wearable-Based Activity Detection

Another method to detect human activities is to use wearable devices, such as wristbands, smart-watches, etc. These devices provide data about the movement of different body parts, heart rate, skin temperature, etc. The activity detection framework collects data from these sensors (features), and applies machine-learning algorithms to classify the observed activities [9]. Depending on the system setup and the activities to be detected, one or more wearable devices are placed on different parts of the body (wrist, ankle, thigh, elbow, hip, chest, etc.) [22]. The classification algorithms used are similar to multimedia-based ones, including support vector machines, Bayesian networks, hidden Markov models, etc.). The advantage of wearable-based systems is that the activity detection can be personalized since the collected data come from a specific person [23]. This is especially useful for health-related applications [24,25]. In addition, the data features have more variety, directly representing the human body conditions and thus can be related to activities more easily.

Mathie et al. [26] used a single waist mounted accelerometer to detect static human activities with 98.90% accuracy. In another work, Gao at al. [27] compared static activity classification performance with a single and multiple accelerometers placed on the chest, waist, thigh and side. Accuracy of a multiple accelerometer system was 6.4% more than that of a single accelerometer. Olguin and Pentland [28] compared accuracy of activity detection across common locations for sensor placement. Placement of data collection modules on the chest or wrist restricts natural movement of the user and makes such implementations even less applicable and useful for applications such as elderly care. Akram et al. [29] used accelerometer data from smartphones to reduce the burden on users of carrying an extra module for data collection. They achieved recognition accuracy of up to 91.15% for six daily activities. The use of smartphone’s acceleration data is also investigated by [30]. Each user performed six activities with a smartphone in their pocket. They evaluated three learning algorithms: logistic regression, J48 and multi-layer perceptron, achieving overall accuracy of more than 90%. Even without the need of a special data collection module, data collection with a smartphone creates dependency on the user, and system performance depends on user cooperation.

The disadvantages of wearable-based activity detection include: (1) obtaining the ground truth might be difficult as the actual activities need to be monitored and cross-correlated with the collected data; (2) the study might be difficult to scale up for multiple people, requiring a lot of devices; (3) the variety of wearable sensors and the fact that the user has to wear or carry them might create discomfort to the user [31], and hence hurting the continuity and longevity of the study; (4) since the wearable devices are user-specific, the data can be easily linked to the user, exposing their identity, and hence creating privacy issues.

### 2.3. Discussion

Multimedia-based and wearable-based solutions provide extremely useful and insightful implementations for human activity detection. However, as we previously mentioned, they result in privacy issues (both), high computation overhead (multimedia), and discomfort issues (wearable). Thus, we believe that, going forward, it will be crucial to address the privacy and discomfort issues by reducing the dependency on the user, and work with data that do not have high pre-processing and processing overhead. Thus, keeping previous research and problems associated with those implementations in the mind, we believe that an ideal system should have the following characteristics: (1) no use of multimedia devices (camera or microphone), (2) sensors embedded within the environment to make accuracy of the system independent of user cooperation, (3) capability to make a few decisions on the less powerful edge device to serve hard real-time operations.

Ambient sensing stands out as a good fit to the above requirements. Ambient sensing is a well-known concept [32], which provides information regarding the surroundings, such as temperature, lighting, pressure, etc. The status of the ambient variables might change due to human activities taking place in a specific environment. For example, the position of a user can be determined because the user might be in front of some ultrasound sensors [33], or the activity of a user can be inferred using a thermal sensor [33]. In all these cases, an activity detection framework does not have any identifying information about the user in the environment, and, since the user does not carry any sensor, the system does not depend on cooperation of users.

In this study, we set up an ambient sensing environment to detect user activities that involve simple movements, such as sitting, standing, and moving left/right. We use two low resolution thermal sensors to detect static and dynamic activities in a single setup, which is non-intrusive, and works completely in real time. In our previous study [33], we showed the feasibility of ambient-sensing based activity detection and showed how this can be done for two simple, static activities (standing and sitting). In this paper, we expand our previous study by adding new static activities, as well as analyzing dynamic activities (that includes constantly moving in the environment). To achieve this, (1) we slightly revise our hardware setup by having an additional thermal sensor, (2) we collect more data for static activity detection, (3) we develop an algorithm for dynamic activity detection, and (4) we present highly accurate results for both types of activity detection.

## 3. Proposed System for Static and Dynamic Activity Detection

Two major concerns we are targeting to solve while implementing our ambient-sensing-based system are that:

(1) The system should satisfy four in-the-wild conditions given in [16] so that natural behavior of human is not restricted.
(2) The system should not violate user privacy. Privacy is always a big concern in sensor based systems [34] where data is stored for future use. Sensors such as camera and microphone raises questions on privacy. We instead are using low-resolution (4 × 16) thermal sensors. With such a small resolution, it is impossible to identify a user, while maintaining non-intrusive, accurate, and real-time activity detection.

### 3.1. Hardware Setup

**Figure 1:**
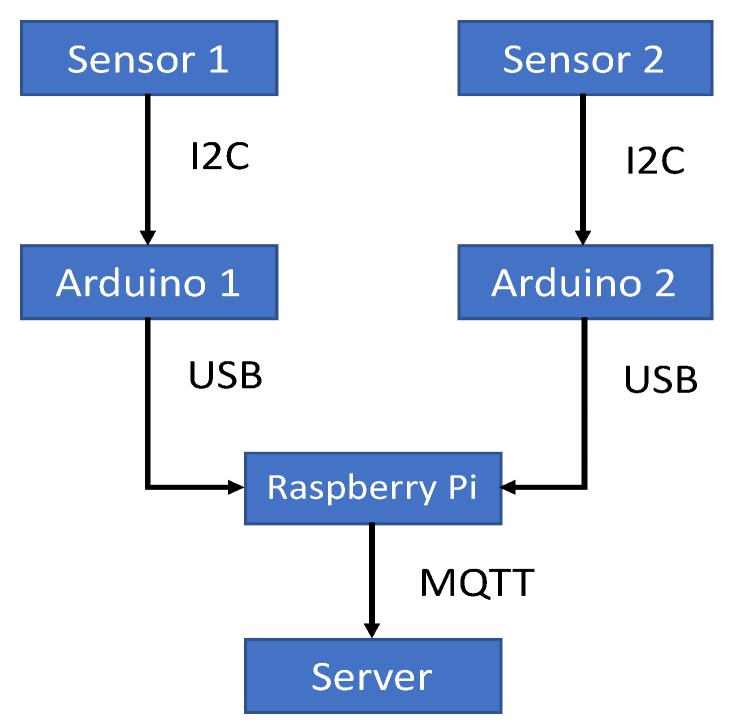
Connection diagram of two sensors to the server.

The basic environmental setup for static and dynamic activity detection remains similar to our previous work [33]. In this setup, we have low resolution thermal sensors (MLX90621, Iper, Belgium) [35], Arduino microcontrollers to process the collected data, a Raspberry Pi to act as a gateway, and a server to represent the cloud. Figure 1 shows the block diagram hierarchical connection and data flow. With this setup, our goal is to create a hierarchical Internet of Things (IoT) hardware and software system, which is shown to be very effective [36].

**Figure 2:**
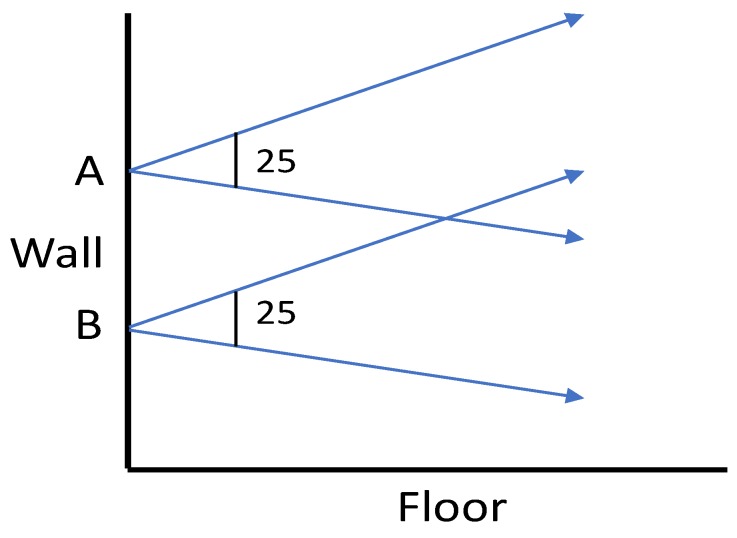
Thermal sensor placement for static and dynamic activity detection. Two sensors are placed on the vertical wall to increase vertical field of view (FOV).

Our thermal sensors are non-contact, and they have 120° horizontal field of view (FOV) and 25° vertical FOV with output in a 4 × 16 array. Since we cannot construct a high-quality image from such a low resolution thermal sensor output, user privacy is not exposed. Each value in the 4 × 16 matrix gives the temperature value in that area which was directly fed to the machine learning algorithms after flattening, without any feature engineering. With two sensors placed on the middle of the vertical wall, as shown in Figure 2, we capture four activities which are standing, sitting on the chair, sitting on the ground, and laying on the ground. These sensors are inexpensive in terms of energy, typically consuming 45 mW of power and can be mounted on perpendicular walls to get wide fields of view.

Two thermal sensors are connected to separate Arduino devices, through I2C interface, and they capture four frames per second. Each Arduino sends data to the Raspberry Pi gateway through USB serial communication. The Raspberry Pi then serializes this data and sends it as a string to an MQTT (Message Queuing Telemetry Transport protocol) broker, where it can be read by our data storage methods. For data storage, we use a mySQL database. The server collects data in real time by reading the data from the MQTT broker using a Python script. This script also saves this data in our mySQL database. This script also calls another Python script which passes the data from the mySQL database (located in the server) to our data analysis models (processed in the server) in the form of a CSV (comma separated values) file. We use MQTT, which is a publish-subscribe based protocol, to publish data to a broker. The database subscribes to this MQTT broker and reads the data and stores it for analysis. The advantage of using the MQTT protocol is that any remote device can subscribe to the broker and read data from it. This enables multiple devices to perform analysis on the collected data.

### 3.2. Static Activity Detection Method

The average temperature of the human body is 36 °C, which is greater than the temperature in a closed environment setting, such as a conference room. A thermal sensor forms an image using infrared radiation and each of its pixels holds the temperature value in that particular region. Static human activities, such as sitting on the chair or standing still, can be captured within a single frame and each activity results in a unique high temperature region in the thermal sensor output. Therefore, the problem of static activity detection is considered a classification problem with each frame of the thermal sensor as input. We compared the performance of six machine learning algorithms. We implemented logistic regression, support vector machine, decision tree, random forest, and naive Bayes algorithms in the open source framework SciKit-Learn, whereas we used TensorFlow to develop the feed-forward neural network method.

**1. Logistic Regression:** Logistic Regression is a simple classification algorithm to predict a discrete variable. It is a strict binary classifier and, in multi-class classification setting, a one-vs.-one or one-vs.-rest scheme is used. We used the one-vs.-rest scheme, a linear solver with L2 regularization strength parameter C=1 in Scikit-Learn.

**2. Support Vector Machine (SVM):** Given a labeled training dataset, SVM predicts the optimal hyper-plane that separates multiple classes. A hyper-plane is a plane separating the space into two half spaces and has dimension one less than the space it is separating. Being strictly a binary classifier, we use one-vs.-rest or one-vs.-one techniques to extend SVM in a multi-class classification setting. We used SVM algorithm from with a third degree *"rbf"* kernel, *"hinge"* loss, one-vs.-one scheme and regularization parameter C=1.

**3. Decision Tree (DecTree):** Decision Tree is a non-parametric, simple to understand machine learning algorithm used for both classification and regression tasks. Being non-parametric, a decision tree makes no assumption about the distribution of the underlying data. Implementation of decision tree in Scikit-Learn uses a CART (classification and regression tree) training algorithm with Gini impurity index.

**4. Random Forest (RandFor):** Ensemble learning algorithms create a set of hypotheses to solve a given problem by training multiple predictors on the given data as opposed to a single hypothesis in other learning algorithms. Ensemble has better generalization ability as compared to its base learner. Random forest is an ensemble method with a decision tree as the base estimator. We used random forest with 10 decision trees.

**5. Naive Bayes (NaiveB):** Naive Bayes is a generative learning algorithm as opposed to discriminative learning algorithms mentioned above. Discriminative algorithms map features to labels, whereas generative algorithms try to predict the distribution of features given a label. We used Gaussian naive Bayes where likelihood of features is assumed Gaussian.

**6. Feed-forward Neural Networks (NN):** The feed-forward neural network is the first and simplest type of artificial neural network built. The information flow happens only in one direction, which is forward, i.e., from the input nodes, across the hidden nodes (if there are any), towards the output nodes. The neurons in the network do not form a cycle or loop [37]. Although single-layer neural networks are popular, they reduce to logistic regression and are capable of capturing only linearly-separable models. Thus, in our work, we built a multi-level feed-forward neural network, specifically a three-layer one. In this network, we have the input layer, which has 128 nodes (we obtain 128 nodes by appending the 4 × 16 output of two thermal sensors in a flattened way, i.e., 2 × 4 × 16 = 128 values). We have two hidden layers, where each layer holds 10 neurons. We implemented this neural network via TensorFlow [38] using its core Python API. Further parameters of a neural network include an activation function (which defines the output of the node) in each node, an algorithm to adjust and learn the weights of each node in each layer, and a learning rate that adjusts the step size of the learning phase [37]. In our study, we used the following parameters: (1) leaky Relu (rectified linear unit) as the activation function, (2) mini-batch gradient descent for learning the weights with batch size 100, and (3) 0.001 as the learning rate of the mini-batch gradient descent algorithm.

### 3.3. Dynamic Activity Detection Method

**Figure 3:**
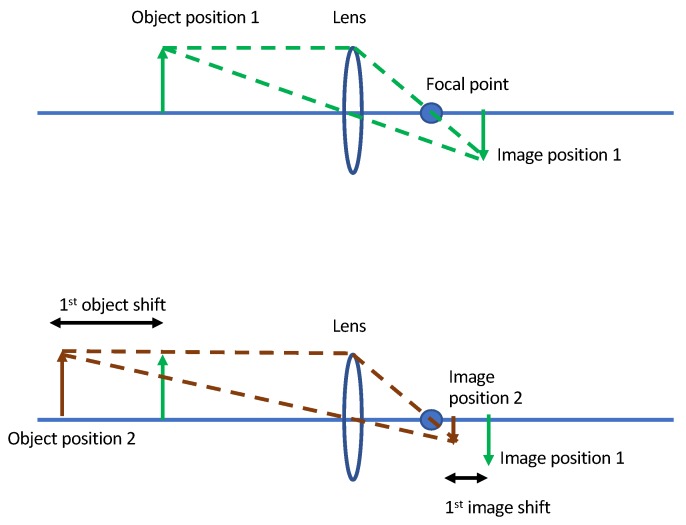
Ray diagram for backward movement with respect to static camera and effect on image captured by lens. Image recreated similar to shown in [39].

Unlike static activities, dynamic human activities such as moving in a particular direction cannot be captured in a single frame from the thermal sensors. Instead, we need to analyze consecutive frames before coming to a conclusion. As a person moves towards the thermal sensor, its distance with respect to the camera lens decreases, increasing size of captured object within the frame. Figure 3 visualizes the above concept using a ray diagram. Extending this concept for thermal images, the size of the high temperature region created due to human presence increases as the distance of humans with respect to camera decreases.

**Figure 4:**
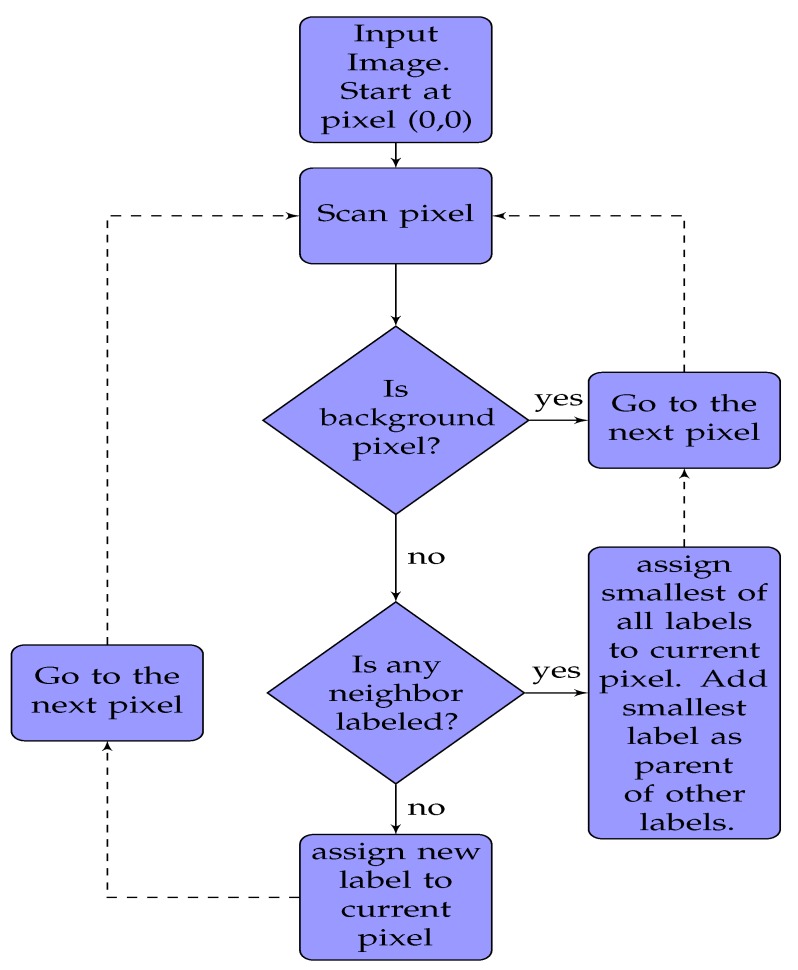
Connected Components algorithm first pass: assigning labels.

**Figure 5:**
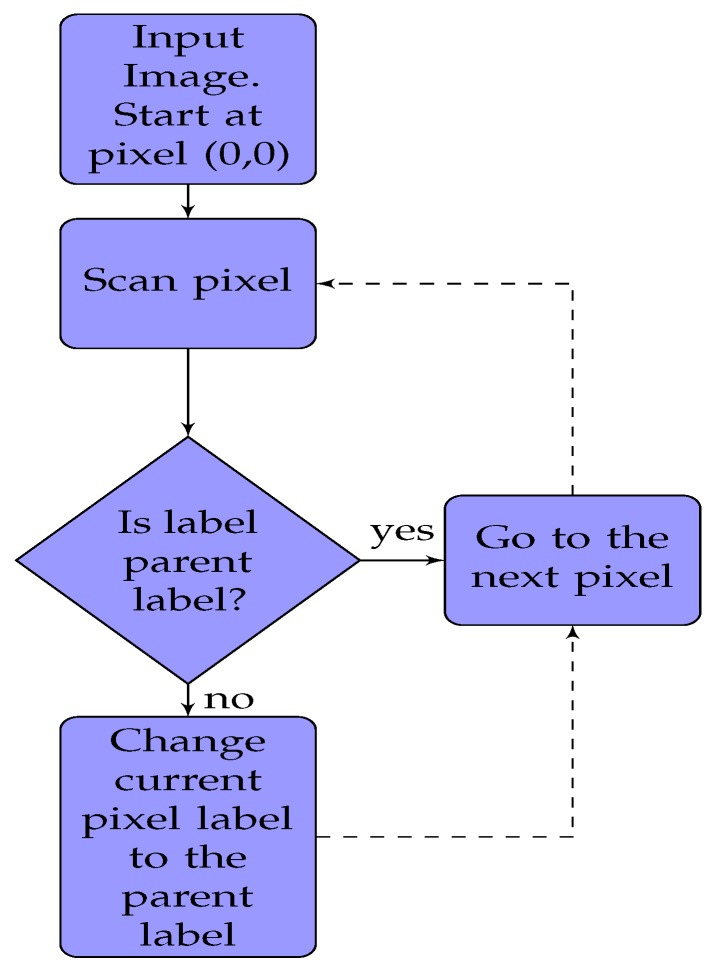
Connected Components algorithm second pass: aggregation.

Connected component labeling [40] is a two pass algorithm to detect connected regions in a binary image. Forward and backward movement of a person with respect to the center frame increases and decreases the number of connected components, respectively. Figure 4 shows an algorithm for the first pass, which is pixel labeling. We start from top the left pixel (0, 0) of an image and scan for non-background (pixel intensity of 255) pixels. For each pixel, we check its neighbouring pixels. If none of the neighbouring pixels is labeled before, we create a new label and assign it to the current pixel. If neighbours of the current pixel are labeled, the smallest of all those labels is assigned to current pixel. However, we also need a way to remember that the current pixel and neighbours are connected, so they cannot have different labels. A union-find data structure is used to store the current pixel label as the parent of the labels of the neighbouring pixels. This information is used in the second pass to clean messy labeling. Figure 5 shows an algorithm for the second pass, which is label aggregation. We scan through an entire image once again starting from the top left (0, 0) pixel. If the label of the current pixel is a parent label, we go to the next pixel. If the label of pixel is not a parent label, the current label is replaced by the parent label, and we move to the next pixel.

Cross-correlation [41] in signal processing is used as a measure of similarity between two series as a function of displacement of one relative to another. The idea of detecting displacement of signals using cross-correlation can be extended to detect displacement of objects given two images. Based on this idea, we take the cross-correlation of the center frame with itself to get the position of highest intensity pixel when person is standing in the center (center is always exactly in front of the thermal sensor). Then, the cross-correlation of each incoming frame with respect to the center gives the shift in the highest intensity pixel from the center. Comparing the position of highest intensity pixel of each incoming frame with its previous frame can be used to determine left–right movement.

## 4. Results

### 4.1. Static Activity Detection

#### 4.1.1. Data Collection

**Figure 6:**
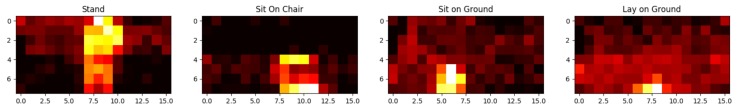
Static activities plotted as heatmap.

We use the non-contact, low-resolution infrared thermal sensors (MLX90621) to collect data for static and dynamic activity detection. Images from infrared image sensor tend to be *Monochrome* because, unlike normal cameras, these do not distinguish between different wavelengths of infrared radiation. A monochrome image, therefore, can be treated as a single channel camera image. MLX90621 has a 120° horizontal field of view (FOV) and 25° vertical FOV, and provides output as a 4 × 16 array. Each value in a 4 × 16 matrix gives the temperature value in the respective area. As shown in Figure 2, we used two MLX90621 with one sensor placed on the top of another sensor. This way, we increase the vertical FOV to capture four static activities: standing (STAND), sitting on chair (SOC), sitting on ground (SOG), and laying on the ground (LOG). Figure 6 shows a sample heatmap of the four activities above.

Each sensor is programmed to get four frames/second with I2C interface connected to an Arduino board. We collected 82,861 labeled examples in seven days with ten volunteers. During data collection, we had the following ensured: (1) there was only one person in the data collection area at a time, (2) there was no object between the sensors and the user, (3) the users could move constantly in the experiment area, and (4) an external observer noted their activities for ground truth. Class-wise distribution of data is as follows: 20,812 (STAND), 20,867 (SOC), 21,182 (SOG), and 20,000 (LOG). In addition, 58,002 examples are used for training and 24,859 examples are used for testing. Each sensor outputs a 4 × 16 matrix of temperature in its field of view. Values from two sensors are appended horizontally to create new 8 × 16 matrix and considered as one frame. These matrices are then fed into the activity detection methods as input.

**Figure 7:**
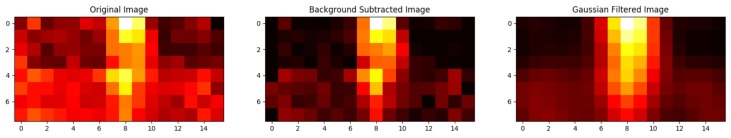
Data pre-processing. (**left**) original thermal sensor frame; (**middle**) background subtracted thermal sensor frame; (**right**) smoothed frame with Gaussian Kernel.

We captured 10,000 background frames and subtracted the average of these frames from each normal frame pixel-wise. For every pixel at location *x,y*, the background-subtracted frame is given by C(x,y)−B(x,y), where *C* is current frame and *B* is the average background frame. A simple background subtraction method works well for this problem because the 4 × 16 array of temperature is a single channel, and change in the light intensity has no effect on the temperature. Finally, we applied a Gaussian filter [42] to each frame before feeding it to the learning algorithm. Figure 7 shows the output of each pre-processing stage. After background subtraction, negative values are set to zero. In Figure 6 and Figure 7, after background subtraction, the dark (black) pixels are zeros. Brightness increases with an elevation in pixel intensity. In our representation, the transformation goes as black -> red -> yellow. Yellow represents the maximum temperature in that region (which is the pixel value of around 255). Therefore, we can say that 0 to 255 in Figure 6 and Figure 7 is mapped from black to yellow (black -> red -> yellow).

#### 4.1.2. Analysis

**Figure 8:**
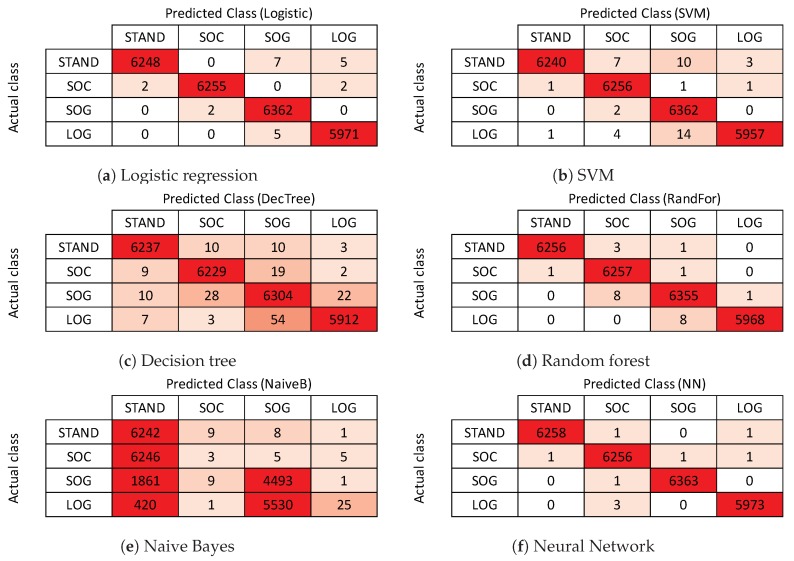
Confusion matrix results for all activities detection using all learning methods.

We split the entire dataset into train and test sets (70% train data and 30% test data). Accuracy is not always a good measure of performance in classification settings [43]. Therefore we used class-wise F1 score along with class-wise and average accuracy values to measure the efficiency of our model. Figure 8 represents the confusion matrices for each method used, where each row represents actual class labels, and each column represents the predicted class labels. Numbers along the diagonal are the correct predictions. *Precision* for each class is the accuracy of positive predictions of the classifier for that class. *Recall*(*Sensitivity*) for each class is the portion of positive instances that are correctly detected by the classifier for that class. F1 score is the harmonic average of precision and recall, having the best value of 1 and the worst value of 0.

- Accuracy = $\frac{T_{P}+T_{N}}{T_{P}+T_{N}+F_{P}+F_{N}},$
- Precision = $\frac{T_{P}}{T_{P}+F_{P}},$
- Recall/Sensitivity = $\frac{T_{P}}{T_{P}+F_{N}},$
- Specificity = $\frac{T_{N}}{T_{N}+F_{P}},$

where $T_{P}$ is true positives, $T_{N}$ is true negatives, $F_{P}$ is false positives and $F_{N}$ is false negatives for given class.

Table 1 shows the average and class-wise accuracy values of all models, whereas Table 2 shows the class-wise F1 score for each model. As shown, the feed-forward neural network performs best at classifying four static activities, resulting in an average accuracy of 99.96%, and F1 score of 99.95%. Gaussian naive Bayes classifier performs worst at static human activity classification tasks with an overall accuracy of 43.29%, and F1 score of 0.88%. Random forest performs slightly less than the best performing feed-forward neural network, giving an average accuracy of 99.90%, and F1 score of 99.92%. Fast train and inference time make them suitable for practical deployment on hardware with low-computational resources. Figure 8 shows the confusion matrices for all the classifiers we used. Specifically, Figure 8a–f correspond to confusion matrices of logistic regression, SVM, decision tree, random forest, naive Bayes, and neural network, respectively. As shown in Figure 8e, naive Bayes classifier gets confused between sitting on ground (SOG) and laying on ground (LOG) activities. As shown in Figure 6, sensor outputs for these two activities are very similar. However, other learning algorithms perform well on these, being able to distinguish between them.

**Figure 9:**
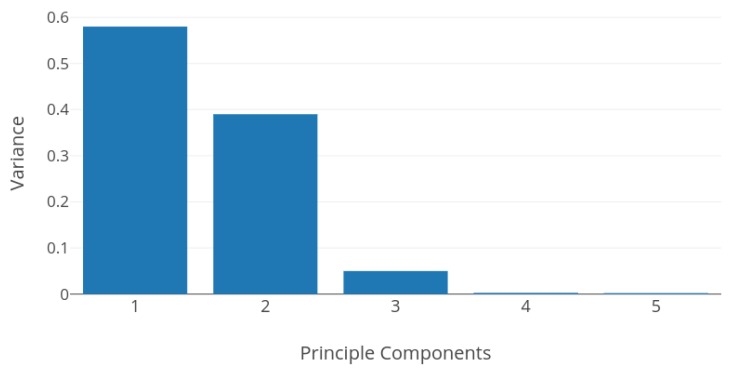
Principle components vs. variance. The first five principle components contribute to more than 98% of cumulative variance.

Logistic regression, which is a simple linear classifier, performs very well on the dataset giving an average accuracy of 99.90%, and F1 score of 99.89%. Insight into *principle components* and *variance* of the dataset can justify the stellar performance of the logistic regression method. Figure 9 shows the first five principle components and their cumulative explained variance. As we can see, the first five principle components account for more that 98% of the variance of the dataset. Pair analyses of these principle components with respect to class labels reveal that principle components for different classes are linearly separable. This is because, after the background subtraction operation and zero clipping, most values in the image matrix are between 0 and 3. Linearly separable principle components explain the good performance of almost all learning algorithms.

**Figure 10:**
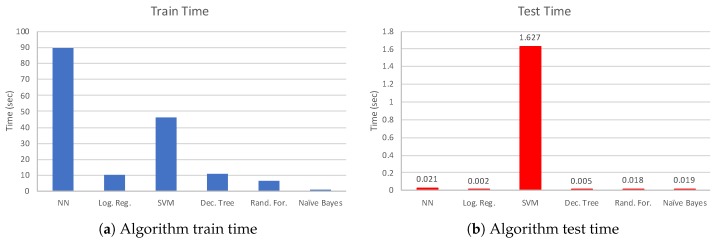
Algorithm train and test time in terms of seconds.

It is also important to know how much time each of these algorithms takes in terms of model training and testing. This determines the suitability of these algorithms in terms of a practical development. For this purpose, we evaluate train (on 70% of entire data) and test time (on 30% of entire data) for all models. In this experiment, we use a workstation with the following characteristics: Intel^®^Xeon^®^CPU E3-1270v5@3.60 GHz processor and 8 GB RAM. This is suitable considering that most of today’s mobile applications rely on cloud computing. Figure 10 shows the results (Figure 10a,b show the train and test time comparisons, respectively). NN (three hidden layers trained in 100 epochs with 10 neurons in each hidden layer) and SVM algorithms have the biggest train time overhead, with 90 and 46 s, respectively. The lowest train time overhead belongs to naive Bayes algorithm due to its simplicity. Logistic regression, decision tree, and random forest have mediocre overhead values (10, 10, and 6 s, respectively). Test times are similar, on the order of 5–20 ms for all the algorithms, except SVM (1.627 s). At this point, considering accuracy performance and test/train times together, we can say that the random forest method provides highly accurate results with low computation overhead at the same time.

#### 4.1.3. Data Collection

**Figure 11:**
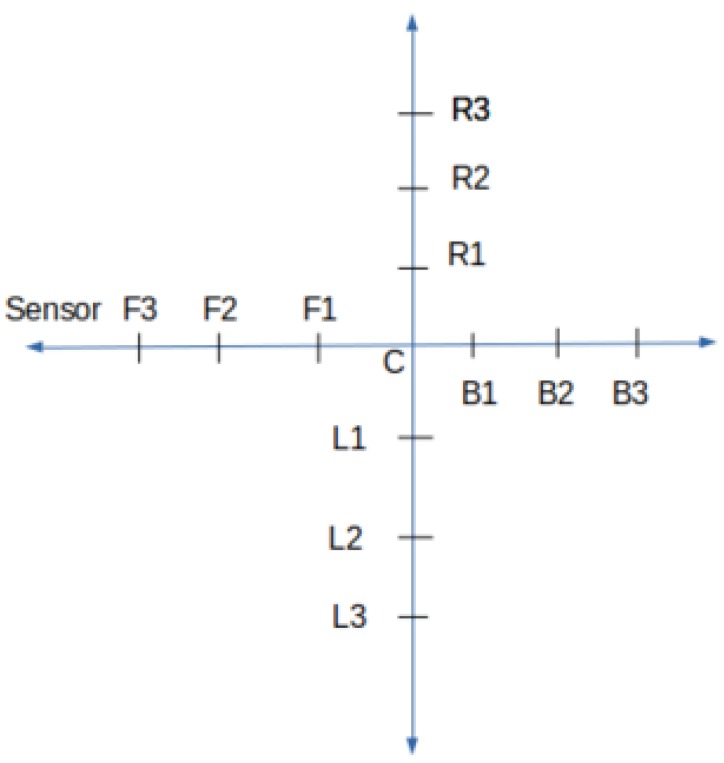
Data collection for dynamic activities. Ri represents point right, Li represents point left, Bi represents point backward and Fi represents points forward with respect to the center.

Data collection for dynamic activity detection is completely different from that of static activity detection. Dynamic activity detection is the detection of forward–backward and left–right movements of users in each frame with respect to a reference frame. A reference frame can be either a previous frame or a fixed center frame in the environment. As shown in Figure 11, forward, backward, left, and right points are marked with respect to the center. Such data collection setup helps to collect continuous data points in each direction. While conducting experiments, the center is kept as a reference point due to two reasons: (1) the size of the test environment is small, leading to smaller distances between two consecutive test points than the average distance covered by a human in a single step, (2) using the last frame as the reference frame does not catch movements from one data point to the next, which is especially the case for cross-correlation, creating bias in the performance metrics. However, in practice, when the size of the environment is big, using the previous frame as a reference frame gives the same result as using the center frame, which we discuss in the next section. This data collection mechanism helps to detect activity for diagonal and movements in other directions, which are not strictly horizontal or vertical. On each data collection point, we collected 600 labeled examples with sensors mounted same as shown in Figure 2. Each collected frame is pre-processed with background subtraction and Gaussian filtering.

**Figure 12:**
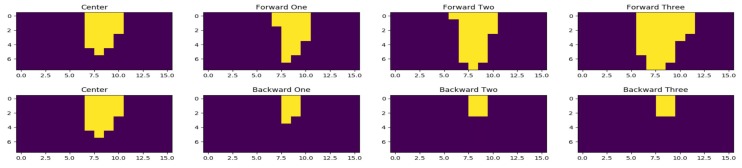
Connected components for forward and backward movements with respect to the center.

**Figure 13:**
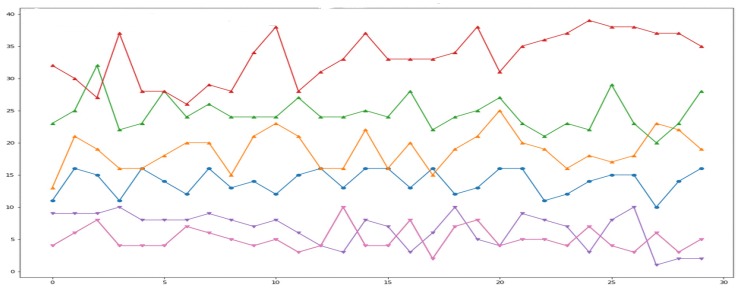
Number of connected components at different data collection points. Blue: Center; Purple: Backward one; Pink: Backward two; Yellow: Forward one; Green: Forward two; Red: Forward three.

We used a connected components labeling algorithm implementation from SciPy [44]. Figure 12 shows how the number of connected components changes as we move forward and backward with respect to the center. Forward One, Forward Two, and Forward Three correspond to data collection points F1, F2, F3 respectively; and Backward One, Backward Two, Backward Three correspond to data collection points B1, B2, B3, respectively, from Figure 11. The first row shows that, as the person moves towards the thermal sensors from the center, the number of connected components increases, whereas the second row shows that the number of connected components decreases, when the person moves away from the thermal sensors. Figure 13 shows the number of connected components plotted for random frames collected at different forward and backward data collection points. The blue line is the plot of the number of connected components for different frames where the person is standing in the center of the environment i.e., at point C. The order of magnitude of Red, Green, and Yellow lines should always be on the order of Red > Green > Yellow followed by the Blue line. This is because the Blue line plots the number of connected components at points [F*i*] going close to the thermal sensors with respect to the center. In contrast, the lines below the Blue line should always follow the order of Purple > Pink. This is due to the fact that they plot the number of connected components of frames collected at data collection points [B*i*] going away from the sensors. Activity detection accuracy with this method is 85.79%, where detection is marked as incorrect if the number of connected components does not follow the increasing or decreasing patterns with respect to the position of previous data collection points for each mark on the *x*-axis. For example, considering data collection points from Figure 11, when we move from F1 to F3, the number of connected components must increase; otherwise, we consider this as an error. In Figure 12 in the second row, as we move from the third column to the fourth, the number of connected components does not decrease, producing an erroneous result.

### 4.2. Dynamic Activity Detection

#### Analysis

We treat the detection of left–right and forward–backward movements as two separate problems. The connected components labeling algorithm is used for forward-backward movement detection, whereas the cross-correlation method is used for left–right movement detection.

**Figure 14:**
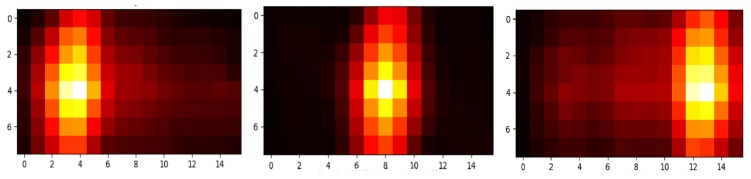
Correlation of each incoming frame with center frame shows shift in the Brightest Spot (BS) with left and right movements. (**Left**) the left position frame correlates with the center position frame: BS(4, 4), (**Center**) the center position frame correlates with itself: BS(8, 4); (**Right**) the right position frame correlates with the center frame: BS(13, 4).

The cross-correlation algorithm implementation we used is also from SciPy. Figure 14 shows the output of three cross-correlation operations as a heatmap and coordinates for the highest intensity pixel. Center frame is the cross-correlation of the center frame with respect to itself, which gives the coordinates of the highest intensity pixel at (8, 4). The left frame shows that the output of the cross-correlation of the center with respect to the frame where the user moves to the left. As we can see, new coordinates for the highest intensity pixel are (4, 4), demonstrating a left movement. The right frame shows the output of the cross-correlation of the center frame with respect to the frame where the user moves to the right. Here, the new coordinates for the highest intensity pixel are (13, 4), showing a movement towards right. Tracking the position of the highest intensity pixel gives us the relative left–right movement of the user in each subsequent frame. This method of cross-correlating each incoming frame with the center frame gives 100% activity detection accuracy for left–right movements and the ability to detect even small movements.

Considering a real-time application and use of cross-correlation on each frame, time complexity of activity detection plays a major role. Convolution is another signal processing method, which gives the amount of overlap when one function is shifted over another. In SciPy, image convolution is computationally less expensive than correlation. Therefore, we used convolution between two images, with one image passed after 180-degree rotation.

**Figure 15:**
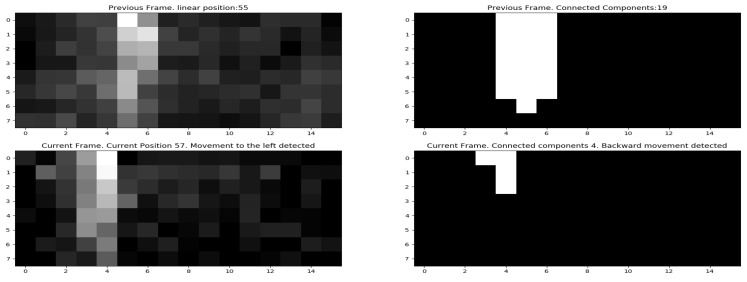
Practical deployment of a dynamic activity detection system working on current and previous frames only.

The dynamic activity detection methods explained above used the center frame as the reference frame to detect activities. As explained in Section 4.1.3, we used this method because the distance between two data collection points is less than the average movement of a human in a single step (which is 2.5 feet for men and 2.2 feet for women [45]). Although necessary in small environments, collecting the center frame is an additional overhead and inconvenient in big environments. We also implemented a system which uses the previous frame as the reference frame, instead of the center frame. We envision that this method could be more suitable for big environments with similar accuracy values. Figure 15, shows a preliminary output of the system deployed for dynamic activity detection using current and previous positions.

## 5. Conclusions

In this paper, we demonstrated the potential use of low resolution thermal sensors for static and dynamic human activity detection in smart environments. Low output resolution of these sensors enables real-time detection due to less computational overhead and mitigates privacy concerns since user identification is almost impossible. For static activity detection, we compared the performance of six machine learning algorithms (logistic regression, support vector machine, decision tree, random forest, naive Bayes, and feed-forward neural network) based on class-wise accuracy and F1 score metrics. Our experimental results show that the feed-forward neural network performs the best with an average accuracy of 99.96%, and F1 score of 99.95%. Performance of the naive Bayes algorithm is the worst (average accuracy of 43.29%) because the feature independence assumption doesn’t hold true in this case. We demonstrate that it is possible to obtain very high accuracy values even for simple learning algorithms, such as logistic regression, as most of the variance in the data set can be explained by the first three principle components of the data. Our computational overhead analysis shows that the random forest method provides a good trade-off between activity detection performance and delay. For dynamic activity detection, the connected component labeling for forward–backward activity detection results in 85.79% accuracy; and the cross-correlation method for left–right movement detection works with 100% accuracy.

## Figures and Tables

**Table 1:** Class-wise accuracy of algorithms (%). Logistic: Logistic Regression, SVM: Support Vector Machines, DecTree: Decision Tree, RandFor: Random Forest, NaiveB: Naive Bayes, NN: Neural Network.

Classifier	STAND	SOC	SOG	LOG	Overall
Logistic	99.94	99.97	99.94	99.95	99.90
SVM	99.91	99.93	99.89	99.90	99.82
DecTree	99.80	99.71	99.42	99.63	99.28
RandFor	99.97	99.94	99.92	99.96	99.90
NaiveB	65.62	74.75	70.17	76.03	43.29
NN	99.98	99.96	99.99	99.97	99.96

**Table 2:** Class-wise F-1 Score of Algorithms (%). Logistic: Logistic Regression, SVM: Support Vector Machines, DecTree: Decision Tree, RandFor: Random Forest, NaiveB: Naive Bayes, NN: Neural Network.

Classifier	STAND	SOC	SOG	LOG
Logistic	99.88	99.95	99.89	99.89
SVM	99.82	99.87	99.78	99.80
DecTree	99.60	99.43	98.87	99.23
RandFor	99.96	99.89	99.85	99.92
NaiveB	53.36	0.09	54.79	0.83
NN	99.97	99.93	99.98	99.95
